# Normal and abnormal development of the aortic wall and valve: correlation with clinical entities

**DOI:** 10.1007/s12471-014-0576-2

**Published:** 2014-07-30

**Authors:** N. Grewal, M. C. DeRuiter, M. R. M. Jongbloed, M. J. Goumans, R. J. M. Klautz, R. E. Poelmann, A. C. Gittenberger-de Groot

**Affiliations:** 1 Department of Cardiothoracic Surgery, Leiden University Medical Center, Leiden, the Netherlands; 2 Department of Anatomy and Embryology, Leiden University Medical Center, Leiden, the Netherlands; 3 Department of Cardiology, Leiden University Medical Center, Postal zone: S-5-24, PO Box 9600, 2300 RC Leiden, the Netherlands; 4 Department of Molecular Cell Biology, Leiden University Medical Center, Leiden, the Netherlands

**Keywords:** Aortopathy, Bicuspid aortic valve, Embryology

## Abstract

Dilation of the wall of the thoracic aorta can be found in patients with a tricuspid (TAV) as well as a bicuspid aortic valve (BAV) with and without a syndromic component. BAV is the most common congenital cardiovascular malformation, with a population prevalence of 0.5–2 %. The clinical course is often characterised by aneurysm formation and in some cases dissection. The non-dilated aortic wall is less well differentiated in all BAV as compared with TAV, thereby conferring inherent developmental susceptibility. Furthermore, a turbulent flow, caused by the inappropriate opening of the bicuspid valve, could accelerate the degenerative process in the aortic wall. However, not all patients with bicuspidy develop clinical complications during their life. We postulate that the increased vulnerability for aortic complications in a subset of patients with BAV is caused by a defect in the early development of the aorta and aortic valve. This review discusses histological and molecular genetic aspects of the normal and abnormal development of the aortic wall and semilunar valves. Aortopathy associated with BAV could be the result of a shared developmental defect during embryogenesis.

## Introduction

Aortic dilation is a pathological widening of the aorta, which can be found in a thoracic (TAD) and abdominal (AAD) form according to its location. In contrast to AAD, TAD is usually not related to atheroma and often occurs at a younger age [1]. Different aetiologies have been described which predispose individuals for TAD, involving monogenic syndromes, such as Marfan (MFS), Ehlers-Danlos (EDS), Smad3 mutations and Loeys-Dietz (LDS) syndromes, sometimes accompanied by bicuspid aortic valve (BAV) as well as idiopathic causes [2], while BAV is also found as an isolated anomaly.

Although patients with isolated BAV may remain asymptomatic, in a significant proportion of the patients the clinical course is accompanied by aortic stenosis, aortic regurgitation, infective endocarditis, and TAD which has a prevalence as high as 50–60 % [3]. Particularly, TAD forms a critical complication, as it carries a risk of dissection and rupture, making it a potentially lethal disease.

Considering these clinical complications, understanding of the development of the ascending aorta and both normal and abnormal aortic valves is mandatory. By sharing a number of embryonic cell types the development of the ascending aorta is narrowly related to the development of the aortic valve. Hence, aortopathy associated with BAV could be the result of a combined developmental defect in early embryogenesis. It has to be kept in mind, however, that not all individuals with BAV develop TAD. In search of the pathogenesis of aortic complications in BAV, the focus has recently shifted towards defining patients susceptible for aortopathy needing aortic intervention.

This review discusses several aspects of normal and abnormal development of the aortic wall and aortic semilunar valves. We hypothesise that the increased vulnerability for aortic complications in BAV is caused by a defect in the early development of the aorta and aortic valve.

## General overview of normal and abnormal aortic valve and aorta development

**Fig. 1:**
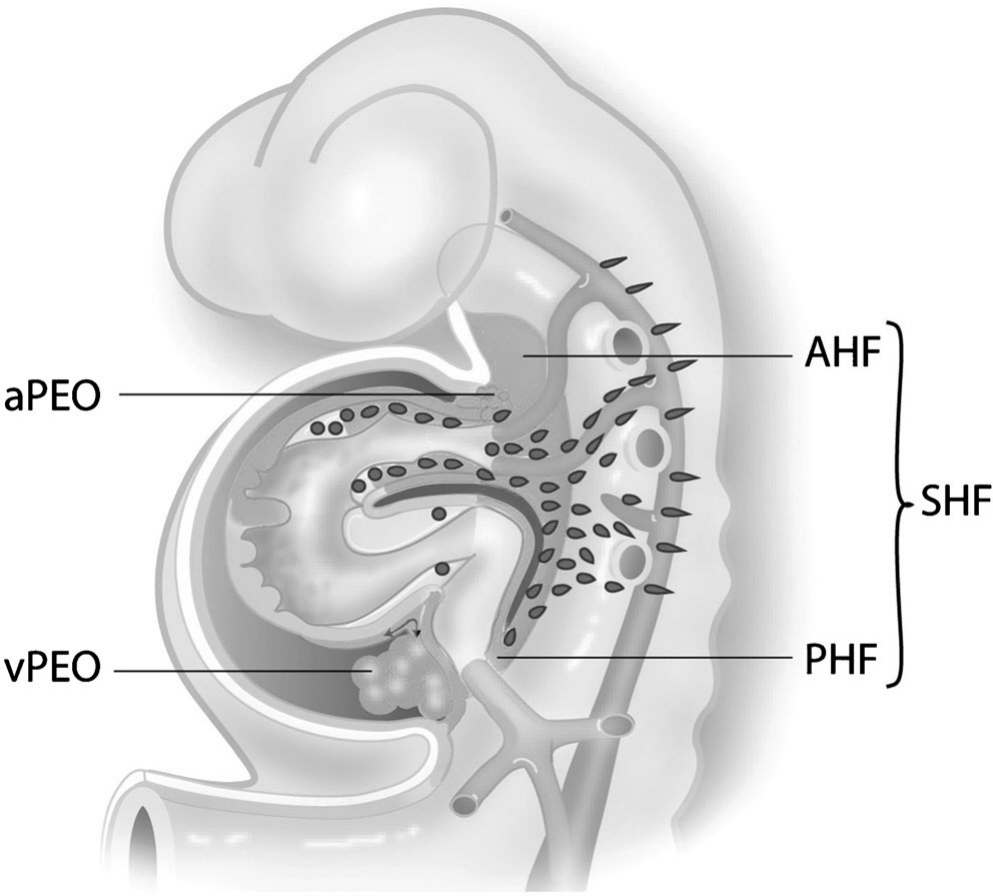
Schematic overview of the developing heart tube. The second heart field (SHF) is indicated contributing to the arterial pole of the heart including the great vessels and the right ventricle by the anterior population (AHF). At the venous pole SHF cells are entering from the posterior population (PHF). Both at the venous (vPEO) and arterial (aPEO) pole a proepicardial organ provides the epicardial cells that cover the myocardium and the intrapericardiac part of the great vessels. Neural crest cells migrate from the neural tube primarily to the arterial pole of the heart

During organogenesis the first functional organ to form is the heart. The first sign of valvulogenesis is the formation of endocardial cushions in the atrioventricular canal and outflow tract. The atrioventricular cushions contribute to the atrioventricular (mitral and tricuspid) valve leaflets, whereas the outflow tract cushions contribute to the semilunar (aortic and pulmonary) valve leaflets [4]. Development of semilunar valves is a complex process in which neural crest cells, second heart field (SHF) progenitors and endocardial cushion derived cells play a role (Fig. 1). The developmental origin of the endocardial cushion cells themselves has been a matter of debate in the past years. Recent lineage tracing studies with Nkx2.5 [5] have shown that SHF progenitor cells give origin to three specific cell lines: 1. vascular smooth muscle cells (VSMCs) of the great arteries, 2. outflow tract and right ventricular myocardium, and to 3. the much discussed endothelial-derived endocardial cushion cells, which are in part derived from the endothelium [5]. Recently, Harmon et al. presented data on the boundary where SHF-derived VSMC meet neural crest cell-derived VSMC at the base of the aorta [5]. The SHF contribution to the aortic media then forms a vertical seam complementary with neural crest-derived VSMC [5]. Next to contributing to the vascular wall, a population of neural crest cells migrates to the outflow tract cushions where they are important for semilunar valve formation and outflow tract septation [6, 7]. Preliminary data show a contribution of the arterial epicardium to the VSMCs of the ascending aorta [8].

During valvulogenesis several signalling pathways such as Wnt/β-catenin, NOTCH, transforming growth factor β (TGF-β), bone morphogenetic protein (BMP), vascular endothelial growth factor (VEGF), NFATc1 and MAPK, as well as transcription factors, including Twist1, Tbx20, Msx1/2, and Sox9, are necessary for the regulation of cell migration, proliferation, and extracellular matrix deposition in the developing valves [9–12].

**Fig. 2:**
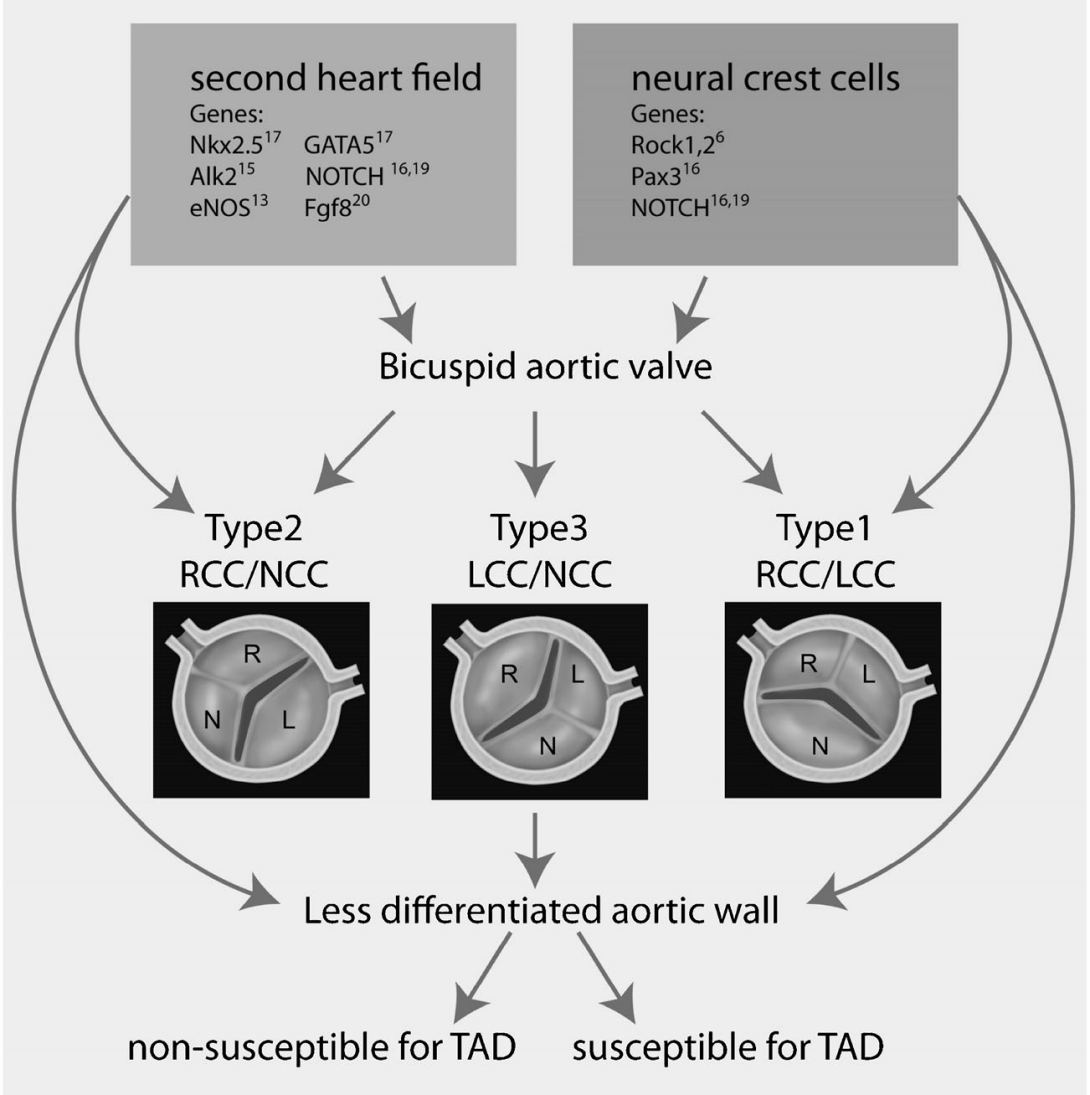
Overview of our hypothesis on the developmental origin of the bicuspid aortic valve and aortic wall abnormalities Figure 2 provides a schematic presentation of the aortic bicuspidy valve types based on the valve leaflet orientation and the position of the raphe. Type 1: with fusion between the right coronary cusp (RCC) and left coronary cusp (LCC). Type 2: with fusion between the RCC and non-coronary cusp (NCC). Type 3 with fusion between the LCC and NCC. Furthermore an overview of our hypothesis on the developmental origin of the bicuspid aortic valve and aortic wall abnormalities is provided. Previously identified genetic mutations in mice (Nkx2.5, Alk2, eNOS, GATA5, NOTCH, Fgf8, Rock1,2 and Pax3) and in human (NOTCH) resulting in bicuspid aortic valves (BAV) are indicated in this figure. These genetic defects can be subdivided in either second heart field (SHF) or neural crest cell related. SHF and neural crest cells both contribute to the vascular smooth muscle cells in the ascending aorta as well as to the cells involved in semilunar valve formation. The SHF most probably also contributes to the endocardial cells of the cardiac outflow tract. Therefore these early developmental defects can cause bicuspidy, but also explain the less well differentiated aortic wall seen in all patients with a BAV. However, not every patient with BAV has increased susceptibility for aortopathy. Therefore an additional factor needs to be identified to recognise patients with increased vulnerability for aortic complications

As a consequence, more than one cell population that contributes to both aortic wall and semilunar valve formation may be involved in the development of bicuspidy. Clinically, several BAV subtypes are distinguished on the basis of the fused commissure or raphe position. Type 1: raphe between right coronary cusp (RCC) and left coronary cusp (LCC), type 2: between RCC and non-coronary cusp (NCC) and type 3: raphe between LCC and NCC (Fig. 2). Clinical outcome differs between the valve types, supporting a different developmental background as underlying cause. Recent support for the role of deficient neural crest cell contribution in development of type 1 BAV was seen in the Rock 1,2 deficient mouse [6]. Fernandez et al. argued that type 1 and type 2 BAVs have a different pathogenesis [13]. An altered neural crest cell behaviour was suggested to be responsible for the development of type 1 BAVs and the endothelial nitric oxide (eNOS) mutation for type 2 BAV. eNOS is expressed by endocardial cells [14], cardiomyocytes [14] and VSMCs [15], all SHF-derived cell types/populations, indicating a role for SHF progenitors in the development of type 2 BAV. Another role for SHF in the development of type 2 BAV was demonstrated by endocardial specific deletion of the gene encoding for the activin type I receptor (ALK2) [16].

Other genetic defects leading to BAV have been identified with selective knockout of genes in murine models but have not focused on the differentiation in type 1 or type 2 BAVs. From the recognised genes, Pax3 is a marker of neural crest cells [17]. Furthermore, the identified SHF markers Nkx2.5 and GATA5 (endocardial cell-specific) are associated with the development of BAV [18].

Abnormalities of NOTCH signalling in the neural crest [19] or SHF can also contribute to the development of abnormal semilunar valves [17, 20]. Interestingly, inhibition of NOTCH in SHF impairs fibroblast growth factor 8 (Fgf8) signalling, which results in the development of BAV, but also in VSMC abnormalities of the great arteries [21]. Therefore, we postulate that a developmental defect of various progenitor cell lines may provide a common mechanism underlying aortic valvulopathy (BAV), as well as aortopathy. The next section focuses on genetic defects described in BAV and TAD in human and their origin in embryogenesis.

## Genetic basis of BAV and TAD

Consistent data have suggested a genetic cause of BAV disease [22, 23]. Despite high heritability it remains challenging to determine the underlying mechanism of BAV in the human population, supported by murine data, as it is probably due to interacting mutations in diverse genes encoding transcription factors, extracellular matrix (ECM) proteins and signalling pathways that regulate cell proliferation, differentiation, adhesion or apoptosis.

Although a remarkable reduction in eNOS levels was seen in BAV patients, this could not be correlated to a mutation in the eNOS gene [24]. Moreover, mutations in the NOTCH1 gene, which is expressed by both neural crest and SHF, and mapped to chromosome 9q34, have been associated with the development and progression of BAV [19, 25]. Further genetic haplotypes within the AXIN1-PDIA2 locus have been recognised that strongly associate with BAV. AXIN1 (Axis Inhibitor 1) is a critical member of the Wnt pathway, which regulates both heart valve formation [26] and cardiac neural crest development [27]. Another haplotype within the Endoglin gene (known as a co-receptor in the TGFβ pathway) is required for differentiation of neural crest cells into VSMCs that populate the aorta [28].

From these genetic defects we conclude that there is a clear link to defects in neural crest as well as SHF-derived cell populations with elements for BAV. Alterations in neural crest signalling are associated with the most common type 1 BAV [29], identified as the valve type with most severe aortic wall abnormalities as compared with the other valve types [13, 30, 31] while SHF-related genes seem to correlate more with BAV type 2.

This brings us to the next important question whether TAD, not necessarily accompanied by BAV, is related to defects in neural crest signalling or alternatively, is there a more specific role for the SHF-derived cells? The following section concentrates on genetics of TAD in syndromes including Marfan, Ehlers-Danlos, Smad3 mutations and Loeys-Dietz and their link to embryonic development.

Marfan syndrome (MFS) is a connective tissue disorder characterised by cardiovascular, skeletal and ocular manifestations. The progressive dilation of the aortic root culminating in dissection is a major cause of morbidity and mortality in MFS patients. This syndrome is the result of a defect in the fibrillin-1 (FBN1) gene that localises on chromosome 15q21.1 and is inherited in an autosomal dominant manner [32]. A second locus for Marfan syndrome (MFS2) has been mapped to chromosome 3p25-24.2, and a heterozygous mutation in TGFΒR2 was subsequently identified as the genetic defect [33]. The TGFBR2 mutations in MFS patients involve the serine-threonine kinase domain and reduce TGFβ-induced receptor signalling.

Loeys-Dietz (LDS) is caused by another defect in the TGFβ signalling pathway. In this syndrome TGFBR1 and TGFBR2 mutations are mapped to chromosome 9q33-34 and 3p24-25 respectively. Cardiovascular lesions in LDS include aortic valve regurgitation and aortic root dilation, aneurysm formation and dissection. Other phenotypic characteristics include craniosynostosis, cleft palate, bifid uvula, congenital heart disease and mental retardation.

Another syndrome presenting with aneurysms, dissections and tortuosity throughout the arterial tree in association with mild craniofacial features and skeletal and cutaneous anomalies has recently been described by Van de Laar et al. [34]. The genetic locus has been mapped to chromosome 15q22.2-24.2 and shows that the disease is caused by mutations in SMAD3, essential for propagation of the TGF-β signal to the nucleus and activation of downstream gene transcription.

An additional syndrome worth mentioning is Ehlers-Danlos (EDS). Patients with vascular EDS often present with dissection or rupture of the thoracic aorta. Aortic dissections have been reported in at least 10 % of patients with EDS [35]. This syndrome is attributed to a mutation in the gene encoding type III procollagen (COL3A1), mapped to 2q24.3-q31 [36]. Type I and type III collagen are the most abundant collagen fibres found in the media and adventitia of the aortic wall. In addition to providing mechanical strength, collagen has other functional properties, including activation of intracellular signalling cascades, storage of soluble factors, such as IL-2, and regulation of their local activity [37].

Furthermore, TAD occurs in association with an autosomal dominant disorder in the absence of syndromic features, termed FTAAD (familial thoracic aortic aneurysm and dissection). A variety of genetic loci have been identified in this regard, such as TAAD1 locus, on chromosome 5q 13-14 [38] and TAAD2 locus on chromosome 3p24-25 [39], the mutant gene associated with this locus is TGFBR2. Only 5 % of investigated families have this mutation, suggesting a relatively rare cause [39]. TAAD3 locus on chromosome 15q24-26 [38, 39] and TAAD4 locus on chromosome 10q23-24 are also related to the development of aortic dissection. In the latter ACTA2 encoding for VSMC a-actin is identified [40]. The mutations impair the function of the VSMCs and this affects the integrity of the vessel wall, making it prone to dilation. This mutation has also been associated with an increased activity of the TGFβ pathway in the aorta [41]. In TAAD5 the identified gene is TGFBR1, mapped to chromosome 9q33-34 [42].

In most of the above TAD cases an increased TGFβ activity has been identified. However, TGFβ is not specific for neural crest, as it is also clearly involved in the endothelium and the SHF-derived cell populations [43]. In syndromes as MFS, EDS, and LDS, bicuspidy is not an obligatory clinical manifestation, indicating that a defective TGFβ signalling is at least not the main factor causing BAV formation. Neural crest defects seem to cause the most frequently occurring type 1 BAV which is associated with most marked complications of the aortic wall, and often with an increased TGFβ activity [44]. However, the clinical course is not complicated with TAD in all BAV patients. Thus, neural crest involvement and TGFβ activity together are not sufficient to explain the variability within the pathogenesis of BAV and associated aortopathy. Additional pathogenetic factors need to be taken into account such as haemodynamics or a contribution of SHF.

## Pathogenesis of TAD in BAV: role of haemodynamics and SHF

The morphology of the bi-leaflet valve produces a nonaxial transvalvular turbulent flow jet within the aortic root [45]. This turbulent flow, along with other haemodynamic factors, as an increased stroke volume (for instance aortic regurgitation), have been suggested to facilitate developing aortic complications, as the created abnormal biomechanics and helical flow alterations lead to an uneven wall stress distribution. However, several studies have confirmed that ascending aortic aneurysms can develop in the absence of valve abnormality [46]. Moreover, Yasuda et al. have reported development of aortic dilation after surgical repair of the diseased bicuspid aortic valve [47]. These studies suggest that structural wall abnormalities at the cellular level may be important for the onset of dilation. Therefore, haemodynamic factors alone are not sufficient to explain the pathogenesis of aortopathy associated with bicuspid aortic valves.

The alternative hypothesis is that genetically determined abnormalities of the aortic wall lead to a defect in the cellular microenvironment, causing or at least contributing to the aortic pathology and render the wall vulnerable to haemodynamic stress.

**Fig. 3:**
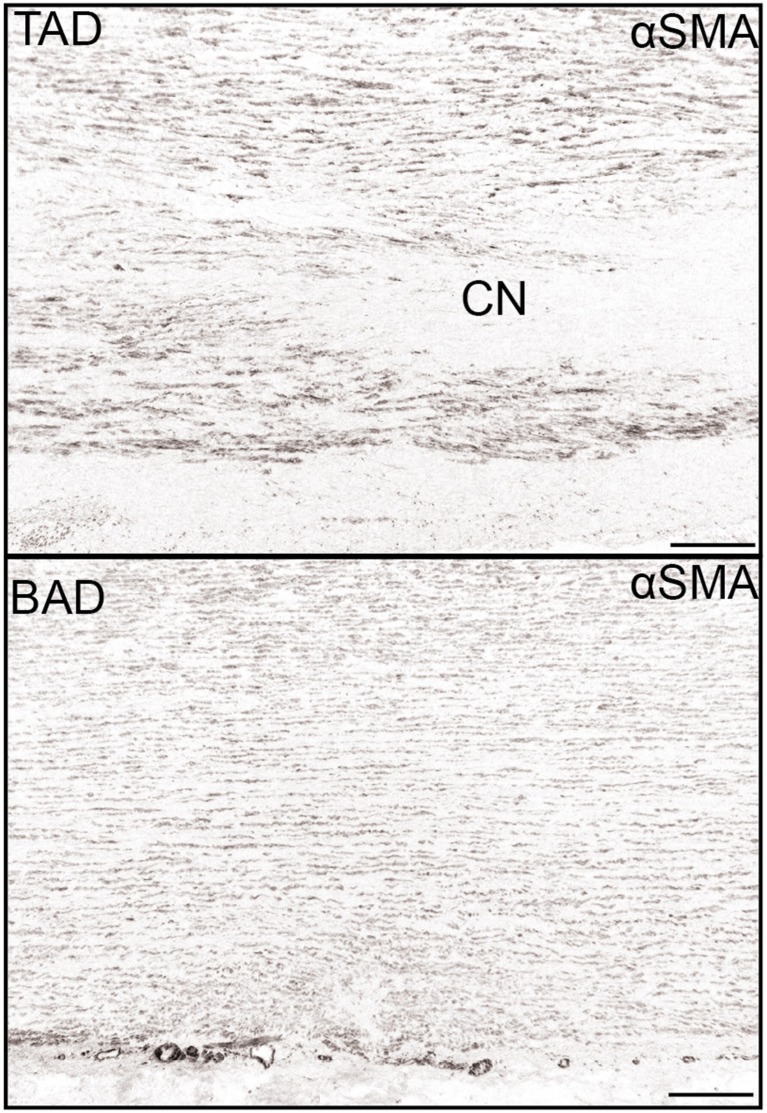
Defective smooth muscle cell differentiations in bicuspidy. Transverse histological sections of the media of the aortic wall stained for the smooth muscle cell marker alpha smooth muscle actin (αSMA). In patients with a tricuspid aortic valve and a dilated aortic wall (TAD) the expression of this marker is higher as compared with the expression in patients with a bicuspid aortic wall and dilated aortic wall (BAD). Furthermore the aortic media in TAD shows significantly more pathology as compared with BAD, with profoundly more cystic medial necrosis (CN) defined as loss of smooth muscle cell nuclei. Magnification bar: 500 μm

Several studies have focused on differences between the dilated aortic wall in BAV and TAV. Histopathological features of the aortic wall in BAV show decreased medial inflammation, elastin fragmentation and cystic medial necrosis, when compared with TAV [48]. In addition, the aortic wall has a different composition in BAV, with a significantly thicker tunica media but significantly thinner tunica intima [49]. In recent years research on ECM composition mainly established differences in the aortic media of dilated aortic wall in BAV and TAV [25, 38]. To take a step further in unravelling a possible different pathogenetic mechanism of TAD in BAV and TAV, we investigated non-dilated aortic walls of both valve types. The aortic media was specifically studied for maturation of VSMCs and ageing characteristics. We concluded that TAD in TAV has aspects of ageing, whereas in bicuspidy there is a defective smooth muscle cell differentiation (Fig. 3) unrelated to ageing. These results suggest that the fundamental difference in the aortic wall make-up of BAV is found in less differentiated VSMCs as compared with TAV while still both neural crest cells and SHF contribute to the VSMCs in the aortic wall. Haemodynamic factors might play a role in the aortic complications, but superimposed on the already present structurally immature aortic wall seen in BAV.

## Conclusion and future perspectives

From a clinical point of view, aortic complications vary between the different BAV types. Aortic root diameters for instance have been analysed between BAV type 1 and 2 in several studies, all of which found larger aortic root diameters in the type 1 [13, 30, 49], being more vulnerable for degradation [31]. Type 2 BAVs are responsible for valve dysfunction at a younger age [30, 49]. Aortopathy seems most outspoken in BAV type 1, probably being caused by a defect in neural crest. Despite these recent findings however, clinical parameters have not been conclusive in distinguishing patients with BAV susceptible for aortopathy, suggesting that an alternative, molecular biological approach might be necessary.

In this review we describe that altered neural crest cell and second heart field contribution, separately or in combination, can account for a structurally different aortic wall in combination with bicuspidy (Fig. 2). Figure 2 summarises that defects in neural crest cells are mostly associated with type 1 BAV and defects in SHF with type 2. These contributions alone, however, are not sufficient to explain the clinical heterogeneity seen in BAV patients, as not all individuals with BAV develop aortic complications during their life. Therefore, additional factors make the aorta susceptible for ensuing complications. It is important to determine which developmental defect accounts for the additional pathology making the aortic wall susceptible for TAD. Future research, therefore, needs to focus on identifying molecular pathways related to neural crest and SHF. These factors are required to distinguish a susceptible and a non-susceptible group for suspected aortic complications.
